# Surface plasmon resonance imaging of cells and surface-associated fibronectin

**DOI:** 10.1186/1471-2121-10-16

**Published:** 2009-02-26

**Authors:** Alexander W Peterson, Michael Halter, Alessandro Tona, Kiran Bhadriraju, Anne L Plant

**Affiliations:** 1 Cell and Tissue Measurements Group, Biochemical Sciences Division, National Institute of Standards and Technology, Gaithersburg, MD, USA; 2 SAIC, Arlington, VA, USA

## Abstract

**Background:**

A critical challenge in cell biology is quantifying the interactions of cells with their extracellular matrix (ECM) environment and the active remodeling by cells of their ECM. Fluorescence microscopy is a commonly employed technique for examining cell-matrix interactions. A label-free imaging method would provide an alternative that would eliminate the requirement of transfected cells and modified biological molecules, and if collected nondestructively, would allow long term observation and analysis of live cells.

**Results:**

Using surface plasmon resonance imaging (SPRI), the deposition of protein by vascular smooth muscle cells (vSMC) cultured on fibronectin was quantified as a function of cell density and distance from the cell periphery. We observed that as much as 120 ng/cm^2 ^of protein was deposited by cells in 24 h.

**Conclusion:**

SPRI is a real-time, low-light-level, label-free imaging technique that allows the simultaneous observation and quantification of protein layers and cellular features. This technique is compatible with live cells such that it is possible to monitor cellular modifications to the extracellular matrix in real-time.

## Background

Cellular remodeling of the ECM is a critical factor in wound healing, developmental biology, metastasis of tumor cells, and diseases such as hypertension [1-4]. The study of cell-matrix dynamics and cellular remodeling of the ECM is challenging, and has involved the use of fluorophores, including fluorescent fusion proteins [5], often using total internal reflection fluorescence microscopy (TIRFM) [6]. We show here that as an alternative, SPRI can be a sensitive, label-free, and low-light optical method that eliminates the requirement for modified biological molecules and transfected cells, and allows for highly sensitive real-time observation of protein deposition and live cell engagement with the ECM.

Surface plasmon resonance (SPR) occurs when light energy couples into the electromagnetic field at a metal-coated surface. The reflectivity of the incident light is inverse to the extent of plasmon resonance, and is determined by the identity and the thickness of the metal layer, the angle of incidence, the wavelength of the incident light, and the refractive index of the medium at the interface. Because the refractive index is proportional to the amount of adsorbate at the surface [7], SPR has been used as a quantitative, sensitive, and label-free technique for measuring the binding kinetics of proteins [8], DNA [9,10], and small molecules [11,12], to surface immobilized capture agents. Using SPR in an imaging mode, high throughput analysis of proteins and DNA has also been demonstrated [13,14]. SPR imaging has not previously been considered a useful technique for imaging cell features, largely because of previous assumptions that poor spatial resolution would prevent useful imaging.

In this report, we demonstrate that SPRI contrast allows sensitive measurement of cell-substrate interactions and mass changes at the substrate interface. SPRI allows quantification of cell secreted and deposited material by observing changes in surface protein mass/area as a function of time and location. We use SPRI to observe the initial surface preparation by monitoring the deposition of the extracellular matrix protein fibronectin which serves as the substrate for the cell based measurements. By using different incident wavelengths and image processing routines for SPRI, it is possible to tune the SPRI measurement for sensitivity versus spatial resolution to suit each step of the experiment. In this report, we demonstrate that SPRI is a sensitive interfacial technique that is able to bridge the gap between molecular (protein adsorption) and cellular (cell-substrate) measurements.

## Results and discussion

### SPRI apparatus and resolution

**Figure 1:**
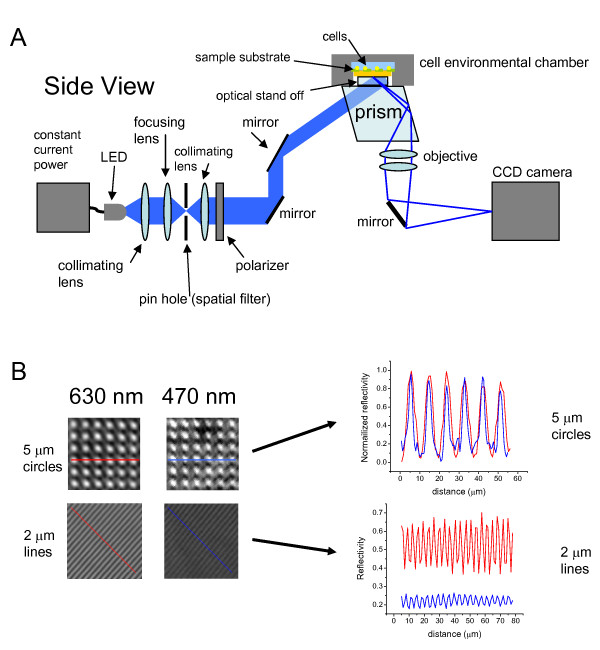
**SPRI apparatus and spatial resolution**. A) Schematic for SPR imaging instrument. Incident LED illumination is spatially filtered, collimated, and directed by mirrors through a SF-10 prism coupled to a commercially available cell environmental chamber designed for transmission and fluorescence microscopies. The reflected image is captured on a CCD camera. B) SPR images were generated with 470 nm and 630 nm incident light. Images shown are 1/16 of the total SPR field of view. Photolithographically patterned PDMS stamps with either 5 μm circle or 2 μm line features were placed directly on bare gold. Normalized intensity line profiles for 5 μm PDMS features compare lateral resolution using 470 nm and 630 nm incident light. Reflectivity line profiles for 2 μm PDMS lines compare reflectance intensity and dynamic range for 470 nm and 630 nm light. Pixel size is approximately 1 μm.

The SPRI apparatus is described schematically in Figure 1A. The optical design is fundamentally similar to existing SPRI instruments [7,15,16], however, the specific configuration is designed to make long term live cell based measurements. By positioning the SPR sensor surface horizontally, incident light is launched from below the sample into a gold-coated SF-10 glass slide which comprises the cell culture surface of an enclosed chamber. This configuration allows cells to be added into the chamber and imaged on the substrate, and facilitates transfer of the cell chamber between the SPR imaging apparatus and an inverted optical microscope. The protocol for SPRI image collection and analysis is described in the Methods section.

Figure 1B provides an indication of the lateral resolution of the SPRI instrument. Polydimethylsiloxane (PDMS) samples, photolithographically patterned with 5 μm circles or 2 μm lines, were placed directly onto SF-10 slides coated with gold and mounting onto the SF-10 prism. The 2 μm patterned lines are arranged obliquely to the direction of the surface plasmon propagation. The SPR images (Fig. 1B) arise due to the difference in the refractive index of air and the refractive index of the PDMS in contact with the surface. Images were acquired using 470 nm and 630 nm incident light as indicated. The 5 μm circle images and line scans are normalized and the normalized reflectivities are plotted displaying a similar spatial sensitivity. The images of the 2 μm lines and line scans are displayed using the raw reflectivity scale to show that the 630 nm SPR image has a larger reflectivity response than the 470 nm SPR image. These results also demonstrate the lateral resolution of the SPRI system to be at least 2 μm based on the ability to distinguish 2 μm features spaced 2 μm apart. This value is in agreement with an estimate made in a previous report using a similar prism based configuration and numerical aperture lens [17].

**Figure 2:**
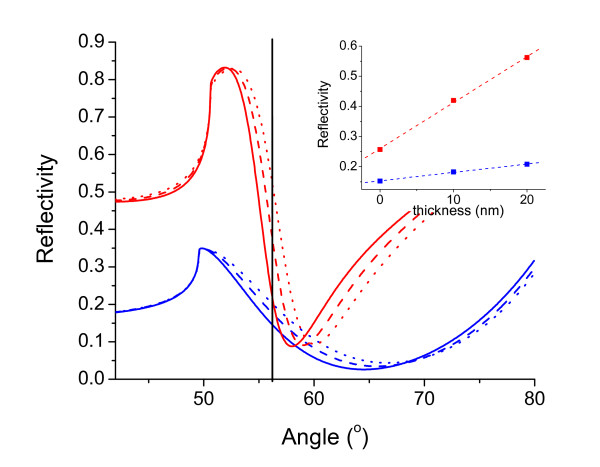
**Linear relationship between reflectivity and refractive index change**. Shown are simulated SPR reflectivity curves of 470 nm (blue) and 630 nm (red) excitation light for 30 nm thick gold. Three curves for each wavelength were used to simulate a protein layer of thickness 0 nm, 10 nm and 20 nm. The vertical line drawn at an angle of 56.0° indicates the incident angle used for SPRI. The inset shows the reflectivity values (at 56.0°) versus protein layer thickness for both wavelengths and shows a linear fit through each. The 630 nm incident light shows a steeper slope than 470 nm for reflectivity change indicating a greater sensitivity to protein adsorption.

Figure 2 shows simulated SPR reflectivity curves to demonstrate the linear relationship between reflectivity and refractive index change. The response is shown for both 470 nm and 630 nm incident light. Reflectivity reaches a minimum when the angle of the incident light is optimal for coupling into plasmons. The SPR reflectivity curves are simulated using published optical constants [18-20] and assuming a four-layer Fresnel model [21] consisting of the prism, the gold layer, adsorbed protein, and aqueous solution that corresponds to the experiments described below. Three curves for each wavelength were used to simulate a compact protein layer of refractive index 1.45 and thickness of 0 nm, 10 nm and 20 nm. The mass/area corresponding to each protein layer is 0 μg/cm^2^, 1.4 μg/cm^2 ^and 2.7 μg/cm^2 ^respectively. SPR imaging is achieved by using a parallel beam of incident light projected onto the surface at a single angle. The SPR imaging angle probed is as indicated by the vertical line in Figure 2. The reflectivity differences at this angle due to the protein layer thickness is displayed in the inset and shows a linear response to mass of material at the interface. Several published reports have also shown a linear relationship between SPR image reflectivity changes and refractive index changes at the surface within a specific range [7,13,16].

In this study, we employed the use of both 470 and 630 nm incident light. According to theory, adjusting the wavelength of the incident light affects spatial resolution, refractive index sensitivity, and evanescent wave penetration depth [22]. However, in practice, we could detect little difference in spatial resolution at the two wavelengths. The main difference was that 470 nm SPR images had a larger depth of field and therefore a larger portion of the image was in focus at the maximal spatial resolution. Also, even though Figure 2 indicates that 630 nm light provided greater sensitivity, the sensitivity at 470 nm was maximized by using a gold thickness (30 nm versus 45 nm) more optimal for 470 nm signal response and image analysis was used to average many pixel values into one measurement. This enabled both wavelengths to be sufficiently sensitive and provide comparable results for protein mass/area. For the data shown here, we used 630 nm incident light for monitoring time-dependent deposition of protein coupled with difference imaging to obtain a signal-to-noise of ~3 ng/cm^2^. For cell measurements we used 470 nm incident light with a signal-to-noise of ~20 ng/cm^2^. Because the cell mass provides a very large SPR signal, the smaller response of 470 nm light helped insure linearity of SPR signal to mass. The SPR evanescent wave penetration depth (defined as decay of the field to 1/e) for 470 nm light is calculated to be ~60 nm compared to ~150 nm for 630 nm light. Thus, about 63% of the intensity of the field for the 470 nm light is penetrating to a depth of 60 nm above the gold surface. The lower penetration of 470 nm light allowed us to image cell-matrix interactions while minimizing contributions to the SPR signal from the cell interior.

### Substrate patterning and fibronectin deposition

**Figure 3:**
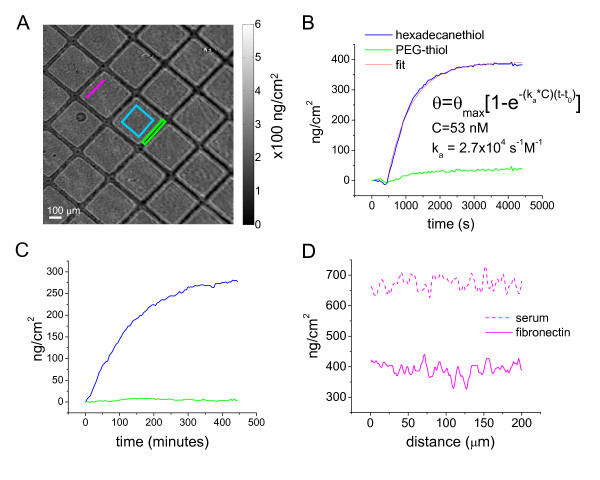
**SPRI kinetics of fibronectin deposition and serum protein adsorption**. A) SPR difference image (630 nm, 56°) after fibronectin deposition onto areas of hexadecanethiol (indicated by the blue square) and PEG-thiol (indicated by the green rectangle). Grey scale values of image intensity display a conversion from reflectivity change (ΔR) to mass/area (ng/cm^2^). B) Kinetic measurements of fibronectin deposition onto hexadecanethiol regions compared to PEG-thiol regions obtained from averaging intensities over the hexadecanethiol and PEG areas, respectively. The observed fibronectin deposition isotherm onto hexadecanethiol fits a basic Langmuir model yielding an association constant k_a _= 2.7 × 10^4 ^s^-1^M^-1^. The solution concentration of fibronectin, C, was 25 μg/mL (= 53 nM). C) Kinetic measurements of relative serum deposition from cell culture medium containing 10% calf serum exposed to fibronectin coated regions compared to PEG coated regions for over 400 min at 37°C. D) 200 μm lines scans (colored magenta in A) on fibronectin coated and serum coated regions. The measured mass/area, in ng/cm^2^, was converted from SPRI reflectivity units using optical properties of globular proteins.

SPRI allows monitoring of the deposition of the extracellular matrix protein, fibronectin, which serves as the ECM for subsequent cell measurements. Microcontact printing [23] was used to prepare 300 μm by 300 μm square patterns of a methyl terminated alkanethiol monolayer on gold. The intervening areas were backfilled with a protein resistant polyethylene glycol (PEG)-terminated alkanethiol, followed by adsorption of fibronectin to the methyl terminated region. Figure 3A shows the resulting SPR image of the pattern of fibronectin areas (lighter squares), each surrounded by darker (thinner) areas of PEG-alkanethiol. Figure 3B shows the kinetics of fibronectin adsorption to the hexadecanethiol coated surface followed by SPRI using 630 nm incident light and difference imaging [15] as described in the Methods. Figure 3B shows that there is little adsorption to the PEG-thiol coated areas. Fibronectin appears to saturate the surface after exposure of about an hour. Because the refractive index changes are directly proportional to mass of protein adsorbed, the rate of protein adsorption and the total protein coverage can be determined (see Methods section). Fitting the data to a homogeneous Langmuir model yielded a rate constant for adsorption of 2.7 × 10^4 ^L mol^-1 ^s^-1^, and a mass/area of approximately 390 ng/cm^2 ^of fibronectin. This value is in good agreement with radiolabeled fibronectin adsorption onto a 1-dodecanethiol coated surface [24]. At saturation, the standard deviation for protein bound was approximately 3 ng/cm^2 ^of protein, providing an estimate of signal-to-noise ratio of greater than 100.

Following fibronectin adsorption, the patterned sample was exposed to serum-containing cell culture medium and measured by SPRI at 630 nm for over 400 minutes at 37°C. The kinetics of adsorption of serum components to the fibronectin coated surface is displayed in Figure 3C and shows approximately 280 ng/cm^2 ^of serum proteins added to the surface at saturation; this result is consistent with a previous report that observed serum albumin adsorption onto fibronectin coated substrates [25]. Negligible adsorption of protein to the PEG-thiol coated areas was observed. The SPRI reflectivity signals were converted to mass/area of serum proteins bound based on the partial specific volume for globular proteins [26,27]. Although SPRI cannot independently identify the specific proteins absorbed, the partial specific volume for globular proteins is highly conserved [28], and therefore the amount of protein adsorbed can be estimated with good accuracy, without requiring specific knowledge of the composition.

Fibronectin and serum proteins appear to adsorb homogeneously onto the hexadecanethiol patterns as shown in line scans of SPR intensity across these areas (Fig. 3D). This observation is shown here to provide contrast to the non-uniform distribution of deposited protein following the addition of cells to the matrix as shown in following sections.

### SPR imaging of cells and patterned fibronectin

**Figure 4:**
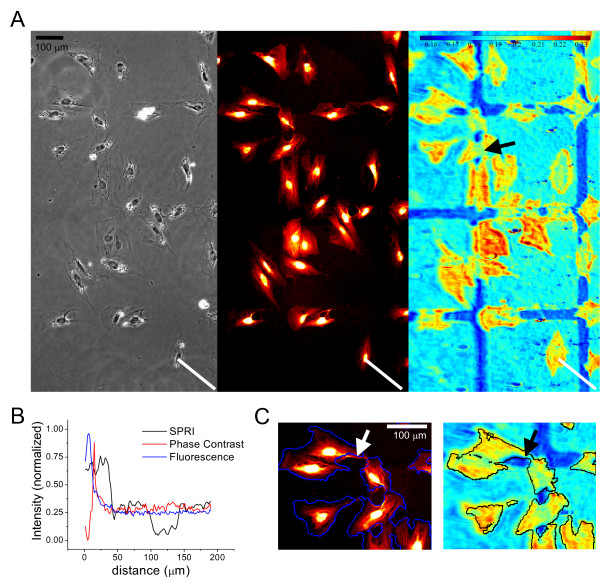
**Comparison of phase contrast, fluorescence and SPR imaging of fixed vSMC 24 h after plating on 300 μm squares of fibronectin separated by 50 μm lines of PEG-thiol**. A) Phase contrast (left frame) and Texas Red fluorescence staining (middle frame) images acquired with a 10×/0.3 N.A. objective on an inverted optical microscope; the SPR image (right frame) was collected on the instrumental set up described herein using 470 nm incident light. The color scale bar indicates reflectivity values. The SPR image displays distinct intensity differences between PEG-thiol regions (dark blue), areas of fibronectin (light blue) and cell-substrate contacts (green to red). The spatial scale bar indicating 100 μm applies to all images. B) Comparison of contrast using the three methods of imaging features under the white lines in (A). C) Selected regions of the fluorescence (left) and SPR (right) images with cell edge contours overlaid from opposing image. Scale bars = 100 μm.

Figure 4 shows the results of cells seeded on the patterned substrates described above. Figure 4A shows an SPR image (right) of vSMC collected using 470 nm incident light and corresponding phase contrast (left) and fluorescence (center) images for the same field of view. The fluorescence image was produced by staining fixed cells with Texas Red maleimide [29]. By comparing the images in Figure 4 it is apparent that of the three imaging modes, only the SPR image allows visualization of both the protein patterned regions as well as the cell-substrate contacts. This highly sensitive discrimination is shown quantitatively in Figure 4B. In each image of Figure 4A, a line is drawn (lower right in all images) that spans a cellular region, a fibronectin region, and a PEG-thiol region; intensities under those lines are plotted in Figure 4B. The line scan for the SPR image shows three intensity plateaus corresponding to each of these regions. The fluorescence and phase contrast line scans, in contrast, show only an intensity change for the cellular regions.

The pseudocolor scale in Figure 4 indicates the reflectivity values associated with areas of different mass. It can be seen that the PEG areas have the lowest mass, with a reflectivity on the order of 0.16. The adsorption of protein to the hexadecanethiol areas results in a higher reflectivity ranging between 0.18 and 0.19, and the greatest mass and highest reflectivity values are associated with regions of cell-substrate interactions. Figure 4 shows that a number of cells appear to span the PEG-thiol regions. Since the cell thickness is much larger than the depth of penetration of the plasmon evanescent wave, SPRI contrast is putatively generated by the distance that the cell membrane is from the surface. Thus, for cells that span PEG locations (Fig. 4A, arrow) the region of the cell above the PEG often has less intensity, suggesting that the part of the cell that spans the PEG-thiol is further from the substrate than the rest of the cell.

The optical contrast provided by SPRI allows label-free segmentation of cell areas. Segmentation of cells (Fig. 4C) based on the SPR signal (right) compared to segmentation based on staining with Texas Red maleimide (left) shows that both SPR and fluorescence images provide similar outlines for cell area. Subtle differences are observed, however, in specific areas that provide a strong fluorescence intensity, but little SPR signal (arrow in Fig. 4C). These observations suggest that portions of cells reside farther away from the interface than is detectable by the SPR evanescent wave.

### SPRI of cellular deposited material

**Figure 5:**
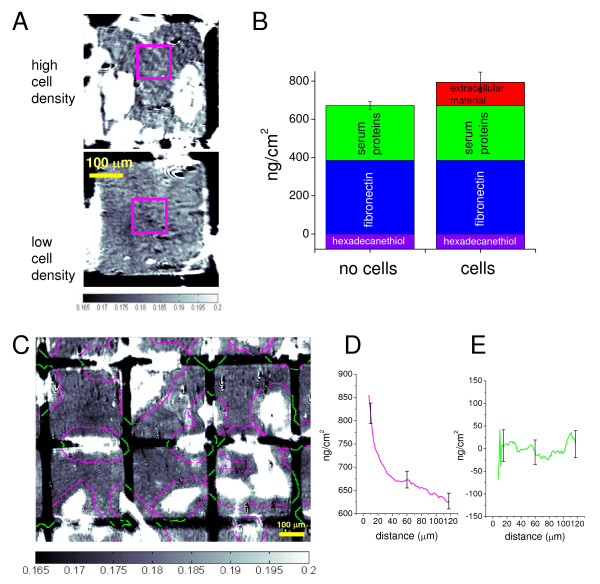
**Quantitation of protein deposition using SPRI**. A) Representative SPR images (using 470 nm incident light) of individual 300 μm squares of fibronectin for regions of relatively high and low cell density. Fibronectin areas are separated by areas of PEG which are resistant to protein adsorption. Image contrast is optimized to aid visualization of differences in protein deposition. The intensity values correspond to reflectivity values shown in the scale bar; reflectivity values > 0.200 appear white and < 0.165 appear black. To determine the amount of protein added to the surface during 24 h in the presence of cells, a 100 μm square region of interest (magenta box) adjacent to cells was selected, and SPR reflectivity within the blue box was averaged and converted to mass/area. A similar region was selected from a region with no cells. B) Bar graphs show that mass of material added to the surface at different steps, and in areas largely devoid of cells and areas containing cells. Mean values and standard deviations are the result from 5 imaged areas. C) Image analysis is used to quantify protein deposition at the cell periphery after 24 h in culture by dilating the cell contours to extract protein mass versus distance from the cell edge in a region where 18% of the area is occupied by cells (approximately 25 cells per field of view). Sequential dilation operations are performed in 1-μm increments. The magenta-colored contours overlay fibronectin regions; green contours overlay PEG-thiol regions. The lines shown around the cells correspond to a distance of 40 μm from the cell edges. Protein coverage in ng/cm^2 ^is determined from the average reflectivity values for each sequential contour. Protein versus distance from cell edge is shown for fibronectin-coated regions (D). The corresponding plot in green is for the PEG-thiol regions (E). The error bars represent the standard deviation from 4 different sample regions. Scale bars = 100 μm. See text for a more complete description of the collection of these data.

It is known that cells modify their extracellular environment and can deposit, attach, secrete, and replace material on the substrate to which they adhere [30-33]. Fluorescence detection can be used to chemically identify individual components in each of these processes. SPRI, in contrast, does not chemically identify specific proteins at an interface, but as we will demonstrate, can be used to generate an overall mass/area measurement of cell-deposited material. The amount of putative cell deposited material attached to the fibronectin patterned surfaces during 24 h of cell culture was quantified by SPRI using 470 nm and 630 nm light. Representative contrast-adjusted images (Fig. 5A) show that protein deposition is greater in fibronectin areas that are more heavily populated by cells (>25% of the area is occupied by cells) than in squares in which there are few cells (<10% of the area is occupied by cells). The SPR signal in 5 regions of high and low cell density were averaged and compared. Using a partial specific volume between that for globular and fibrillar proteins, we determined that, on average, an additional 120 ng/cm^2 ^of protein was associated with regions of high cell occupancy versus low cell occupancy. The PEG-thiol areas remained essentially unchanged during this time. A schematic of the layers of protein deposition detected by SPRI is shown in Figure 5B. Approximately 390 ng/cm^2 ^of fibronectin adsorbed onto hexadecanethiol, 280 ng/cm^2 ^serum proteins adsorbed onto fibronectin, and an additional 120 ng/cm^2 ^protein was deposited onto the fibronectin patterns at regions of high cell density. The layer schematic does not account for possible displacement of fibronectin by serum proteins, which has been observed to be minimal on hydrophobic substrates [25].

Unlike the adsorption of fibronectin and serum proteins from solution, which was largely spatially homogeneous as shown in Figure 3D, the deposition of additional protein after cell seeding was not uniform. Spatial analysis of cell-derived protein was performed by determining the amount of protein deposited as a function of spatial proximity to cell edges. SPR images of fibronectin-coated areas occupied with varying densities of cells are shown in Figure 5C. Cell edges were defined with a single threshold value, and used to create a contour outline of each cell, which was then sequentially dilated in 1-pixel steps (1-pixel = 1 μm) out to 120 μm from the cell edge (Additional File 1). The image processing used to generate these images is described in Methods. In Figure 5C, the contour line is shown at 40 μm from the cell edge, and is colored magenta when it overlays a fibronectin area and green when it overlays a PEG-thiol region. The SPR intensity values under the magenta or green lines were averaged for each contour trace around the cell periphery. Figure 5D shows the plot of deposited protein coverage versus distance from the cell edge for fibronectin coated areas. The amount of material on these regions decreases with distance away from a cell. This observation is consistent with the idea that more cell-derived protein would be deposited closer to cells than further away. The high surface coverage of fibronectin used in this study (390 ng/cm^2 ^= 5200 molecules/μm^2^) was chosen to minimize cell migration [34]. Hence, we surmise that most of the material detected is likely cell-secreted and/or cell-assembled. Notably, no measurable protein deposition onto the PEG-thiol areas was detected (Fig. 5E, which is a plot of the data depicted by green lines from Fig. 5C). These data demonstrate that the difference in reflectivity with distance-from-cell edge is due to material that deposits onto fibronectin but not onto PEG.

While the flat response of the PEG distance-to-cell-edge coverage plot in Figure 5E shows that no measurable cell derived material is being deposited onto the PEG regions, it also serves as an image analysis control to insure influence from edge effects is minimized. This also shows that other effects such as lateral decay of SPR signal are not observed to bleed into low signal regions.

We also observed that in areas of low cell density (<15% of area corresponding to cells) and high cell density (>25% of area corresponding to cells) that there was deposition of protein that was dependent on distance from cell edges (Additional File 1). This distance dependence was observed to be more pronounced when applied to regions of lower cell density. The sometimes more subtle distance dependence at high cell density is perhaps due to the influence of material secreted by neighboring cells.

**Figure 6:**
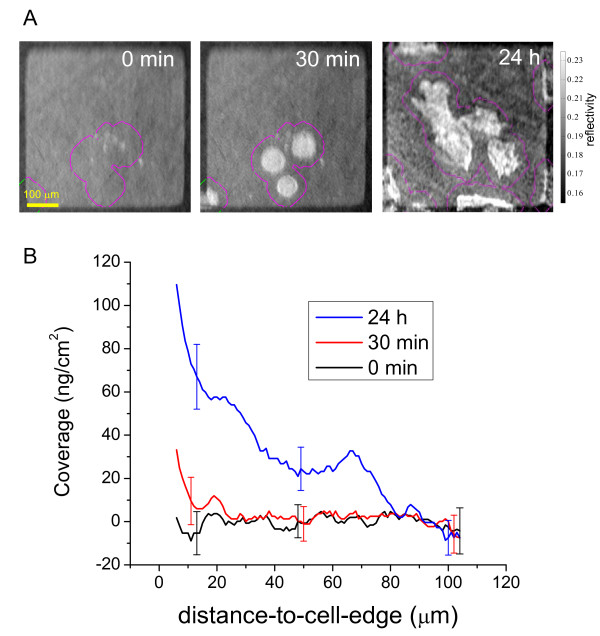
**Image analysis of cell deposited material at cell periphery of live cells**. A) Three time-lapse images of cell spreading on a 500 μm × 500 μm fibronectin patterned substrate separated by PEG that has been pre-incubated with serum medium exposure overnight. The times indicate 0 min, 30 min and 24 h after addition of cells to the chamber. Each image is overlaid with contour outlines depicting the distance-to-cell-edge coverage analysis routine (described in text) shown here at 20 μm from cell edge. For the 0 min starting time frame, the distance-to-cell-edge analysis routine used is the same as that for the 30 min time frame. B) The image extracted coverage versus distance-to-cell-edge data is displayed for the image at each time point. The starting mass/area is scaled to begin at 0 ng/cm^2^. The early time points (0 min, 30 min) show relatively even spatial distribution of surface coverage. The 24 h time point shows a mass distribution of putative cell-deposited material that occurs proximal to cell edge. Error bars represent the standard deviation from 4 different sample regions.

To assess the time-dependent deposition of cellular protein, Figure 6 shows analysis of the addition of protein to the substrate by live cells at different time points (Fig. 6A), These data were generated using the approach applied to the data in Figure 5. Cells were identified by simple thresholding as described earlier, and this threshold provided indication of the cell edges. In this experiment, cells were seeded onto 500 μm × 500 μm fibronectin patterns that had been pre-incubated with serum-containing media overnight to allow serum proteins to adsorb to the fibronectin areas. Immediately after addition of cells, no additional material is observed to adsorb onto the fibronectin substrate and the surface coverage is observed to be evenly distributed spatially (Fig. 6B). At 30 min after addition of cells, increased SPR signals, indicating the presence of newly adsorbed protein, appear in areas close to the cell edges. At 24 h after seeding, additional protein mass is detected and is distributed spatially as a function of proximity to the cell edge. Similar to the analysis on fixed cells, most of the cell-derived additions to the substrate appear within 40 μm of the cell edges. This suggests that this additional material is derived from cells, and could be contributed to by cell secretions, cell material left behind following lamelopodia retraction, and/or cell assembly of ECM proteins from the serum.

**Figure 7:**
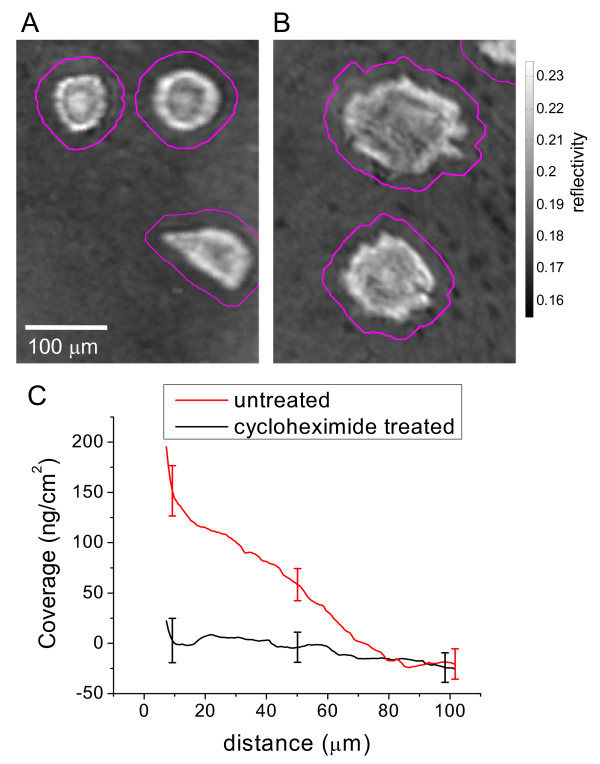
**Image analysis comparison of cell deposited material at cell periphery for cycloheximide treated cells compared to untreatedcells**. A) Representative SPR image of vSMC 24 h after plating and exposure to cycloheximide. B) Representative SPR image of untreated vSMC 24 h after plating for comparison. Each image is overlaid with contour outlines depicting the distance-to-cell-edge coverage analysis routine (described in text) shown here at 20 μm from cell edge. C) The image extracted coverage versus distance-to-cell-edge data is displayed for vSMC that are treated and untreated with cycloheximide. The data represent the average of 5 different 500 μm by 500 μm fibronectin patterned regions and the error bars represent the standard deviation.

As verification that the additional additive material is completely cell derived, vSMC were plated in growth medium containing 100 μg/ml cycloheximide (CHX), a protein synthesis inhibitor [35]. The approach allows us to observe the interaction of the cells with the fibronectin patterned surface in the absence of new protein synthesis. Figure 7A shows a representative image of CHX treated cells that are fixed 24 h after plating and Figure 7B shows an image of untreated cells. The data were generated using the approach applied to the data in Figure 5. At 24 h after plating, additional mass is detected proximal to the cell edge for the untreated cells but no additional material is observed to adsorb onto the fibronectin surface for the CHX treated cells (Fig. 7C). From this, we conclude that the additional material is derived from cells.

## Conclusion

Our results show that SPR images, obtained without the addition of fluorescent or other labels, can provide information on cell-substrate contacts, ECM deposition and substrate protein patterns. Cells can be observed over long periods of time because cellular damage is minimized at the low light levels that are sufficient for SPRI. A previous report demonstrated the first example of SPR imaging of cells [36], but the present study describes how the use of appropriate optical architecture, light sources, and image analysis permit a successful application of SPRI. This study demonstrates the use of SPRI to perform cell object segmentation, protein pattern identification, and quantitation of protein deposition by cells. Live-cell measurements by SPRI demonstrate the time-dependent deposition of cellular protein. The data indicate that SPRI can detect deposited protein with a sensitivity of ~20 ng/cm^2^, and can achieve a lateral resolution of at least 2 μm. While the current study does not attempt to identify the proteins that cells contribute to the matrix, future integration of SPRI with fluorescence based microscopy methods will combine the unique label-free information from SPRI of live cells with complementary biochemical information for a more complete understanding of cell interactions with their extracellular environment.

## Methods

### Substrate preparation

SF-10 glass slides (25 mm × 25 mm × 1 mm) (Schott, Elmsford, NY) were acid-washed with 7:3 (v/v) H_2_SO_4_:H_2_O_2_, rinsed with 18 MΩ cm distilled water, rinsed with ethanol, dried under a N_2 _stream, and then coated with ~1 nm chromium and ~30 nm gold by magnetron sputtering using an Edwards Auto 306 vacuum system (Edwards, Wilmington, MA) at 1 × 10^-7 ^mbar. We selected this thickness to be optimized for 470 nm incident light instead of thicker gold (45 nm would be optimal for coupling 630 nm light) in order to maximize its transmissivity and facilitate the phase contrast and fluorescence measurements.

Polydimethylsiloxane (PDMS) stamps with 300 μm or 500 μm square features spaced 50 μm apart were used for microcontact printing. Masters for casting PDMS, (Sylgard 184, Dow-Corning, Midland, MI) stamps were made using a dry film resist technique and PDMS stamps were cast from these as previously described [37]. Microcontact printing of hexadecanethiol to the gold substrate was performed according to a published procedure [23] using a 2 mM hexadecanethiol (Aldrich, St. Loius, MO) solution in ethanol. Following stamping with hexadecanethiol, the slides were immersed in a 0.5 mM solution of HS(CH_2_)_11_(OCH_2_CH_2_)_3_OH (PEG-thiol)(Asemblon, Seattle, Washington) in ethanol for 12 h in order to coat the exposed gold with protein resistant polyethylene glycol terminated alkanethiol. The hexadecanethiol areas of the patterned substrates were coated with fibronectin by inserting slides into a sterile solution of 25 μg/ml bovine plasma fibronectin (Sigma, St. Louis, MO) in Ca^2+^-and Mg^2+^-free Dulbecco's phosphate buffered saline (DPBS; Invitrogen, Carlsbad, CA) for 1 h to 2 h. For the SPRI kinetics experiment, an alkanethiol patterned gold slide was mounted into an environmental cell culture/viewing chamber (FCS2; Bioptechs, Butler, PA), rinsed by flowing through DPBS, and placed onto the SPRI device. A solution of 25 μg/ml fibronectin in DPBS was flowed through the chamber at 1 mL/min, stopped, and the kinetics of deposition monitored by SPRI for 1 h at room temperature.

### Cell culture, fixing, and staining

The rat aortic vascular smooth muscle cell line (vSMC) (A10; ATCC, Manassas, VA) was maintained in Dulbecco's Modified Eagles Medium (DMEM; Mediatech, Herndon, VA) supplemented with nonessential amino acids, glutamine, penicillin (100 units/mL), streptomycin (100 μg/mL), 10% by volume FBS (Invitrogen, Carlsbad, CA) and 25 mM HEPES, and maintained in a humidified 5% CO_2 _balanced-air atmosphere at 37°C. Cells were removed from tissue culture polystyrene flasks by trypsinization, and were seeded in culture medium onto the fibronectin patterned substrates at a density of 2000 cells/cm^2^. After 24 h incubation, cells on the substrates were washed with warm Hanks Balanced Salt Solution (HBSS; ICN Biomedicals, Costa Mesa, CA), fixed in 1% (v/v) paraformaldehyde in DPBS (30 min) at room temperature, quenched in 0.25% (m/v) NH_4_Cl in DPBS (15 min) and rinsed with DPBS. Cells were permeabilized and stained for 1 h with Texas Red-C_2_-Maleimide as described previously [29] (10 mg/mL in DMF stock, dissolved in DPBS (1 mg/mL) containing 0.1% (v/v) Triton X-100). After rinsing with DPBS, the fixed cell substrates were mounted into an environmental chamber (FCS2, Bioptechs) and kept under DPBS for all microscopy measurements. For the SPRI kinetics experiments with live cells, cells were seeded by gravity flow onto the fibronectin patterned substrate that was already mounted in the environmental chamber, and kept at 37°C for the duration of the experiment.

### SPRI apparatus

A SPR imaging instrument was built to provide a horizontal imaging surface for ease of compatibility with custom and commercially available cell chamber devices (Figure 1). An adjustable constant current power supply (Wavelength Electronics, location, Bozeman, MT) was used to power high candela LEDs (THC3; LSDiodes, Lake Oswego, Oregon) of wavelength 470 nm +/- 5 nm (blue) or 630 nm +/- 10 nm (red). The incoherent light was collimated, spatially filtered through a pin hole, recollimated, passed through a linear polarizer (Newport, Irvine, CA), and directed to the prism imaging surface at the incident angle of 56° by two broadband dielectric mirrors (Thorlabs, Newton, NJ). The environmental flow chamber was assembled with a gold-coated SF-10 glass slide; this slide serves as the substrate which was prepared as described above. The cell culture chamber was mounted on top of a SF-10 rhomboid prism (Optical Fabrication Shop, NIST, Gaithersburg, MD). An 'optical stand-off' (10 mm × 10 mm × 1 mm SF-10 glass slide) was designed to bridge the gap (~1 mm) between the bottom of the FCS2 Bioptechs chamber window with the top of the SPR prism. SF-10 index matching fluid, n = 1.725 (Cargille Laboratories, Cedar Grove, NJ), was used to optically couple each interface. The refractive index of the SF-10 glass is greater than the refractive index of the medium above it, and the geometry is configured for total internal reflection. The incident light couples into the plasmons of the gold film, resulting in a fraction of the p-polarized light being absorbed and fraction of the light being reflected as a function of the refractive index of the medium, the angle of incidence and the wavelength of light [22]. The reflected SPR image was collected by two achromatic lenses (Thorlabs) arranged in the configuration of a 10× Lister objective [38] with a working distance of 30 mm and a calculated NA = 0.38. According to theory, this imaging objective is estimated to resolve <1 μm features. The image was reflected off a mirror onto a 12-bit (2048 pixel × 2048 pixel) Retiga 4000 CCD camera (Qimaging, Surrey, Canada). Images were collected with Qimaging software.

### SPRI collection and analysis

For each SPR image, both p- and s-polarized light images were taken by manually rotating the linear polarizer 90°. Only p-polarized light interacts with the surface plasmons while s-polarized light remains proportional to the incident light. Dividing p- by s-polarized light provides a normalized value for reflectivity, and also normalizes for spatial inhomogeneity in incident light. For each static image a 630 nm and 470 nm wavelength excitation SPR image was obtained. For the kinetics of fibronectin deposition, a 630 nm p- and s-polarized image was taken at the beginning and end of the experiment while only 630 nm p-polarized light was used for the kinetics. Regions of interest (ROI) were selected on the hexadecanethiol and PEG-thiol areas (Figure 3) and the kinetics of deposition were monitored by 'difference imaging' in which the change in reflectivity is measured by subtracting the first time point image from all subsequent images.

For live cell time-lapse images, a 470 nm p- and s-polarized image was taken at the beginning and end of the experiment while only 470 nm p-polarized light was used for the time-lapse imaging. Live-cell time-lapse images presented in Figure 6 are displayed as reflectivity intensity values as indicated by the intensity scalebar.

SPR imaging suffers from two types of image distortion that is rarely discussed in the SPR imaging literature [39] but is addressed in the Brewster angle microscopy literature [40,41] which also is dependent on imaging based on an oblique angle of the sample relative to the collection optics. The first is image skew in one axis of the x-y plane of the image; the second is out of focus regions in the image because the object plane is not parallel to the image plane. We use image processing to correct for image skew by dividing the y-direction of the image (the direction of lateral plasmon propagation and image illumination) by 1/cos(θ) where θ = 56° for the angle of incident illumination. We limit analysis to the center portion of the image that is in focus.

A previous report details the conversion of SPRI reflectivity changes into mass/area coverage [7]. In that study, a calibration factor for the SPR system is necessary. Here, we use the relation, θ = sΔR, where mass/area (in ng/cm^2^) (θ) is directly proportional to the change in reflectivity (ΔR) and multiplied by a system calibration constant (s). The calibration constant is determined from the fibronectin deposition experiment assuming that the effective thickness of a fibronectin layer deposited onto a hexadecanethiol monolayer is 3 nm [42] and the partial specific volume of fibronectin [43] is 0.73 cm^3^/g. All subsequent coverage values reported (in ng/cm^2^), in the case of serum proteins adsorbed or putative cell deposited proteins, assume a partial specific volume 0.73 cm^3^/g. This value is in good agreement to values for many globular proteins [26,27], and fibrillar proteins such as collagen [44].

All image analysis was performed using custom and stock code written in MATLAB and MATLAB Image processing toolbox (Mathworks, Natick, MA).

### Image analysis routine for protein deposition at cell periphery

**Figure 8:**
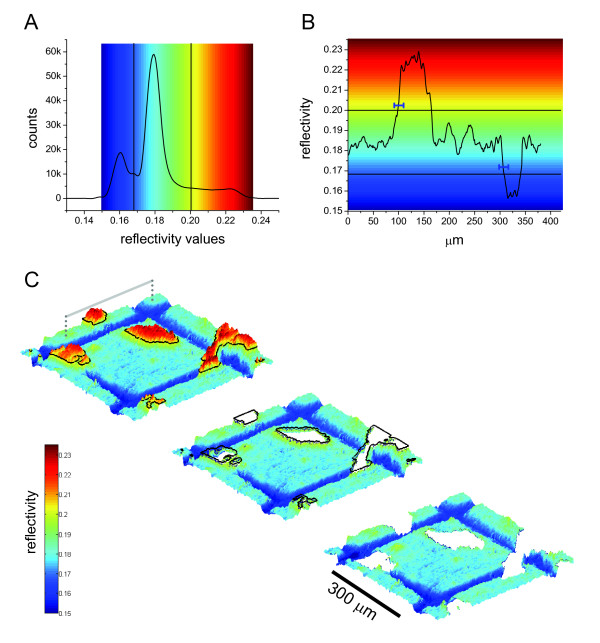
**Illustration of the image analysis procedure to quantify protein deposition at cell edge**. A) A histogram of image reflectivity pseudocolored with the colorbar scale shown is divided into three distinct regions: PEG-thiol (dark blue), fibronectin/protein (light blue-green), and cell features (yellow-red). B) A line that spans the regions of cell contact, fibronectin/protein layer, and PEG-thiol layer is shown as the grey bar in (C) and the reflectivity values under that line is shown in (B). The horizontal blue bars mark the width of the transition (average 5 μm to 8 μm) from one region to the other. C) Sequential image analysis steps for a SPR topographic image of a 300 μm square fibronectin island where the z-axis corresponding to reflectivity values shown in the colorbar. The first image shows the cell, protein, and PEG-thiol regions with a black ring segmenting the cell-protein threshold, at a reflectivity value of 0.20, as displayed in (A) and (B). The second image deletes the cell objects and the black line highlights the initial cell contour ring that still borders part of the cell edge as seen in (B). The third image dilates the cell contour by 5 pixels clearing the cell-protein transition region as shown in (B). It is at this point that sequential contour dilating to determine surface protein coverage surrounding cells is begun. The black scale bar equals 300 μm, and the gray bar corresponds to the line scan used in (B).

The image analysis routine applied above was developed to extract coverage information as a function of cell-edge proximity and the analysis protocol is detailed here (Fig. 8). 1) Object segmentation of the 470 nm SPR image into three sections: PEG, protein, and cell features, was performed manually based on the three peak values of the image histogram (Fig. 8A). The histogram threshold values selected were reflectivity = 0.167 for the PEG to fibronectin edge, and reflectivity = 0.200 for the fibronectin to cell edge. The histogram obtained threshold values were compared with a representative line scan (Fig 8B) and agreed with the results of edge segmentation. The representative line scan revealed that the transition width between cell-to-protein regions, and protein-to-PEG regions occurred over a range of 5 to 8 pixels (represented by the blue bars in Figure 8B). 2) Binary masks were created for each of the object sections. 3) The image mask for cell features was dilated 5 pixels, and the protein and PEG masks were eroded by 5 pixels to avoid any edge transition effects in subsequent analysis. Figure 8C depicts this process for cell features using a topographic view of SPRI reflectivity of a cell on a 300 μm square fibronectin pattern. Here, the images step through the process of cell segmentation to mask dilation and shows these values subtracted from the original image. 4) Using the dilated cell mask, the mask was dilated 1 more pixel and then subtracted from the previous cell mask resulting in a 1 pixel wide ring encircling the cell structure. 5) The ring mask was multiplied by the protein and PEG mask and the resulting mask was plotted on top of the original image as magenta-colored if the ring overlaid the fibronectin pattern or green if it overlaid the PEG region as shown in Figure 5C. 6) Numerically, each resulting mask was multiplied by the original image and all of the fibronectin overlaid reflectivity values were averaged as well as all of the PEG-thiol overlaid values. 7) The process of cell contour expansion was repeated in 1 pixel increments and data was collected as reflectivity intensity vs. pixel distance from cell edge. 8) The reflectivity values were converted to coverage values (ng/cm^2^) as described in the section above and the pixel values were converted to μm (1 pixel = 1 μm).

### Fluorescence and phase contrast microscopy

Phase contrast and fluorescence microscopy images were acquired using a 10×/0.3 NA objective on a Zeiss Axiovert 200 inverted microscope (Carl Zeiss MicroImaging, Inc., Thornwood, NY) and a CoolSNAP HQ CCD camera (Roper Scientific, Tucson, AZ). Flatfield images generated with a 1 μL droplet of a 50% fluorescein solution in 0.1 mM NaHCO_3 _between a microscope slide and coverslip [45] were used to correct for inhomogeneity of the fluorescence illumination. Because of the larger field of view afforded by the SPRI setup, images from two adjacent fields of phase contrast and fluorescence microscopy were stitched together to be compared to the corresponding SPR image (Fig, 4A). The SPR image was registered to the phase image using 6 fiduciary marks. The SPR incident wavelength, 470 nm, and fluorescence excitation wavelength (555 nm) for Texas Red staining were chosen so as not to have an overlapping excitation.

## Authors' contributions

AWP, MH, AT, and KB performed the experiments. AWP performed data analysis. AWP, MH, and ALP prepared the manuscript. All authors read and approved the final manuscript.

## Supplementary Material

Additional file 1 **Quantifying protein deposition at cell periphery**. Movie showing image analysis procedure used to quantify protein deposition at the cell periphery after 24 h in culture by dilating the cell contours to extract protein coverage versus distance-from-cell edge. Also, shows image analysis for low (<15%) and high (>25%) cell density regions. Click here for file
